# The Synergistic Effects of Combined Use of *Mentha longifolia, Thymus carmanicus*, and *Trachyspermum copticum* on Growth Performance, Feed Utilization, and Expression of Key Immune Genes in Rainbow Trout (*Oncorhynchus mykiss*)

**DOI:** 10.3389/fvets.2021.810261

**Published:** 2022-01-14

**Authors:** Mehdi Raissy, Mehdi Ahmadi Kabootarkhani, Kimia Sanisales, Mohammad Mohammadi, Ghasem Rashidian

**Affiliations:** ^1^Department of Aquatic Animal Health, Faculty of Veterinary Medicine, Shahrekord Branch, Islamic Azad University, Shahrekord, Iran; ^2^Faculty of Veterinary Medicine, Shahrekord Branch, Islamic Azad University, Shahrekord, Iran; ^3^Department of Aquaculture, Faculty of Natural Resources and Marine Sciences, Tarbiat Modares University, Noor, Iran

**Keywords:** medicinal plants, combination of plants, non-specific immunity, immune genes, growth performance, rainbow trout

## Abstract

Medicinal plants exhibit remarkable positive effects on different aspects of fish physiology. This study aimed to evaluate the possible impact of a combination of plants (*Mentha longifolia, Thymus carmanicus*, and *Trachyspermum copticum*) on growth performance, immune responses and key immune gene expression of rainbow trout. For this purpose, four diets were designed, including zero, 0.25, 0.5, and 1% of a mixture of plants per kg of diet, representing dietary treatments of control, T1, T2, and T3, respectively. Two hundred forty fish (weighing 23.11 ± 0.57 g) were fed 3% of body weight twice a day for 45 days. The results showed that growth parameters of weight gain (except for T1) and FCR were significantly improved in fish receiving all levels of plants, with T3 showing the best growth results. Digestive enzymes activities were notably increased in T1 and T2 compared to the control. Stress biomarkers (glucose and cortisol) were significantly decreased in T1 and T2, while T3 was not significantly different from the control. Immunological responses were significantly improved in T2, while T1 andT3 did not show a statistical difference in terms of lysozyme activity. Catalase activity was noticeably decreased in T1, although superoxide dismutase and malondialdehyde were highest in T2. Immune-related genes were significantly up-regulated in T3 compared to other treatments. Also, antioxidant enzyme coding genes were strongly up-regulated in T2 and T3. Overall, the present results suggest that 1% inclusion of the mixture of *M. longifolia, T. carmanicus, and T. copticum* (T2) can be used to improve the growth and immunity of rainbow trout.

## Introduction

Aquaculture has been exponentially growing during recent decades. However, sustainable development of aquaculture is hindered by several influential factors among which infectious disease has led to economic losses and indiscriminate use of antibiotics (1). In aquaculture, prophylactic measures are extremely important to secure high-quality fish production. The fish innate immunity plays a crucial role in this scenario, and thus the potential of immunostimulants, including medicinal plants (raw material/extracts/essential oils), have been extensively studied aiming to improve growth and immunity of fish (2–4). The principle of selecting plants for such purposes relies on their traditionally known therapeutics and their main bioactive substances. In addition, plant-based products are less expensive with fewer side effects.

Rainbow trout (*Oncorhynchus mykiss*) from the *Salmonidae* family is of economic importance with 916,540 tons of global production in 2019 (5). Several plants have been reported effective on growth performance, digestive enzymes, and immune responses of rainbow trout (6–9). However, few studies have investigated the combined use of plants.

*Thymus carmanicus* and *Mentha longifolia* belong to the family *Lamiaceae* and contain high amounts of polyphenolic compounds contributing to their antioxidative activities (10–14). *T. carmanicus* has been reported to contain large amounts of Linalool, carvacrol, and thymol as major components (15). *M. longifolia* is wildly spread in vast areas of Europe and Asia with a long history of application in traditional medicine to treat gastrointestinal disorders, cold, fever, spasm, and flatulence (16). The major components present in *M. longifolia* are pulegone, isomenthone, 1,8-cineole, borneol, and piperitenone oxide (17). A recent study demonstrated 4% to 6% inclusion of *M. longifolia* extract was effective on the growth and immunity of Caspian kutum (*Rutilus frisii kutum*) (18). In addition, Heydari et al. (12) have suggested that 0.2% and 0.3% are substantially effective on the immune system of rainbow trout and the expression of TNF-α was remarkably increased in fish that received the extract of *M. longifolia*.

*Trachyspermum copticum* (Ajowan) belongs to *Apiaceae* family, with its oval and yellowish fruits as well as its aerial parts (flower and leaves), is used as a diuretic, carminative, and anthelmintic (19). Ajowan has been reported to be a significant antiviral (20), anti-inflammatory (21), antimicrobial (22, 23), and antifungal (24). The main active substances of Ajowan are *P*-Cymene, γ-Terpinene, Thymol, β-Pinene, and Myrcene (24).

The current study was designed to assess the possible effects of different dietary levels of combined plants of *Thymus carmanicus, Mentha longifoliai, Trachyspermum copticum* on digestive enzyme activity, growth performance, biochemical, immune and antioxidant parameters of serum, and expression of immune and antioxidant-related genes in rainbow trout.

## Materials and Methods

### Collecting Plants

The seeds of *Trachyspermum copticum* and leaves of *Thymus carmanicus* and*, Mentha longifolia* were procured from a local market. The plants were identified at the Research Center for Medicinal Plants and Ethno-Veterinary, Islamic Azad University, Shahrekord, Iran. All plants were fresh and collected in 2021.

### Experimental Diets

Four different diets were prepared to assess the effects of the plant mixture on rainbow trout's growth performance and immunity. All plant materials were dried, finely powdered, mixed together in equal amounts, and kept in the refrigerator until use. The experimental diets were formulated to contain different levels of zero, 0.5, 1, and 2% of the mixture of plants per kg diet referred to as control, T1, T2, and T3 respectively. In summary, desired levels of a mixture of powdered plant materials were added to the diet ingredients (Table 1) and vigorously mixed with enough water. Then the paste was cooled at 4°C and passed through a grinder. The pelleted feed (3 mm) was dried at room temperature for 48 h, sieved (3 mm) and finally coated with 1 ml of fish oil to prohibit leaching. The feed was kept at 4°C, in clean plastic bags until use.

**Table 1:** Diet formulation of experimental diets incorporated with different levels of zero (Control), 0.5% (T1), 1% (T2), and 2% (T3) of a mixture of herbs (*Thymus carmanicus, Mentha longifolia, Trachyspermum copticum*).

**T3**	**T2**	**T1**	**Control**	**Ingredients (%)**
34	34	34	34	Fish meal
2	1	0.5	0	TMT^1^
17.5	17.5	17.5	17.5	Corn gluten
18.0	18.0	18.0	18.0	Wheat flour
4.8	4.8	4.8	4.8	Sunflower oil
14.0	14.0	14.0	14.0	Soybean meal
3.6	3.6	3.6	3.6	Rice bran
2.3	2.3	2.3	2.3	Fish oil
1.5	1.5	1.5	1.5	Vitamin premix^2^
1.5	1.5	1.5	1.5	Mineral premix^3^
2.0	2.0	2.0	2.0	Molasses
0.03	0.03	0.03	0.03	L-Carnitine
0.70	0.70	0.70	0.70	Salt
0.02	0.02	0.02	0.02	Vitamin C

1 *Thymus carmanicus, Mentha longifolia, Trachyspermum copticum*;

2 *Vitamin premix: retinol acetate (A), 6000 IU; Cholecalciferol (D3), 2250 IU; DL-atocopheryl acetate (E), 225 mg; menadione sodium bisulfite (K3),15 mg; L-ascorbic acid (C), 700 mg; Dbiotin(H2), 0/6 mg; thiamin mononitrate (B1), 36 mg; riboflavin (B2),45 mg; calcium D-pantothenate (B3), 7200 mg; niacin amide (B5), 135 mg; pyridoxine hydrochloride (B6), 36 mg; folic acid (B9), 9 mg; cyanocobalamin (B12), 0/045 mg; antioxidant 75 mg*,

3 *Mineral premix: mineral: Fe, 45 mg; Cu, 5/4 mg; Co, 0.75mg; Se, 0.15 mg; Zn, 75 mg; Mn 5/37 mg; I, 5/4 mg; cholinechloride, 2250 mg*.

### Feeding and Culture Conditions

Rainbow trout were secured from a nearby provider and were secured for up to two weeks to adjust to research facility conditions. During this period, the fish were bolstered a basal diet (Kimiagaran. Co, Iran). Afterward, 240 healthy fish (23.11 ± 0.57 g) were distributed in 12 tanks and fed 3% of their respective biomass twice daily for 45 days. During the experiment, tanks were aerated using air stones and uneaten feed was removed from the tanks. Physiochemical parameters of rearing water including temperature, pH, Dissolved Oxygen and hardness (CaCO_3_) were measured as 17 ± 1°C, 7.52 ± 0.22, 7.2 ± 0.39 mg.ml^−1^ and 164.9 ± 18 during the experiment. The photoperiod was set as 12L:12D using artificial light.

### Sampling

The sampling procedures were in accordance with our previous publication (25) with slight modifications. Blood samples were drained from six randomly selected fish per replicate after 24 h starvation. Fish were anesthetized with clove powder (50 mg.ml^**–**1^), blood was drained from the caudal vein using 2 ml heparinized syringes, then samples were left to clot at room temperature, centrifuged at 3,000 rpm for 10 min, and kept at −20°C until further investigation. Three fish were randomly selected for collecting skin mucus, placed in small plastic bags holding 5 ml of saline buffer (0.09% NaCl) for 1 min while gently rubbing fish from the operculum toward the tail. Three fish were euthanized using an overdose of clove powder (200 mg.ml^−1^) and dissected in an aseptic condition. The liver and intestine were snap-frozen in liquid nitrogen for gene expression studies. Three fish were also euthanized in the same manner, and the whole digestive tract was extracted to measure digestive enzymes' activity. The complete digestive tract was thoroughly washed with distilled water then homogenized using an electric homogenizer (IKA T25 digital, Ultra Turrax model) on ice flakes in 10 volumes (w/v) of cold physiological serum. Subsequently, samples were centrifuged at 6,000 *g* for 20 min at 4°C. The supernatants were then stored at −80°C in new tubes until further evaluation.

### Growth Parameters

At the end of the experiment, growth parameters of final weight (precision of 0.01 g), weight gain (WG), specific growth rate (SGR), feed conversion ratio (FCR), and survival rate (SR), were measured based on the following equations:


$$\begin{array}{l} \text{ WG (g)}=\text{Final weight – Initial weight};\\ \text{ FCR}=\text{Total Feed Given (g)/Weight gain (g)};\\ \text{SGR (\%.}{\text{d}}^{-1}\text{)}=\text{(\{Ln final wt (g) – Ln initial wt (g)\}/days)}\\ \;\times 100;\\ \text{ SR (\%)}=\text{(final numbers/initial numbers)}\times 100. \end{array}$$

### Digestive Enzymes Activity

Protease was evaluated using the casein hydrolysis method (26). The absorbance of the supernatant was valued at 280 nm beside L-tyrosine that was used as standard. The amount of enzyme that secreted one mmol of tyrosine ml^−1^ min^−1^ was determined to be one unit of protease activity. The method described by Bernfeld (27) was applied to measure amylase activity. Maltose was used as the standard substance, and absorbance was read at 600 nm. The amount of enzyme that produced one mmol maltose ml^−1^.min^−1^ was determined as one unit of amylase activity. Lipase activity was evaluated based on the assessment of enzyme hydrolysis, diacylglycerols, triacylglycerols and monoacylglycerols to free fatty acids in olive oil emulsion. The hydrolysis of 1.0 micro equivalent of fatty acids from triacylglycerol in 1 h with a pH of 7.7 and temperature of 37°C was selected as one unit of lipase activity.

### Assessment of Serum Biochemical Parameters

Serum total protein, albumin, triglyceride, glucose, cortisol and cholesterol were measured by Pars Azmun kits (Pars Azmun, Iran) in line with the kit's instructions. The activity of alkaline phosphatase (ALP), aspartate transaminase (AST) and alkaline transaminase (ALT) were evaluated calorimetrically at the wavelength 540 nm using ready-made kits (Pars Azmun, Iran) under the kit's guidelines.

### Serum Innate Immune Responses

The recommended protocol by Ghafarifarsani et al. (28) was followed to measure lysozyme activity. In summary, 175 μl of *Micrococcus luteus (*Sigma, M 3770, St. Louis, USA) suspension (75 μg.ml^**–**1^) was prepared in 0.1 M phosphate citrate buffer (pH 5.8) mixing it with 25 μl of fish homogenate samples. A microplate reader (Hiperion, Germany) was used to record the changes in turbidity at 450 nm for a continuous 5 min. The quantity of lysozyme leading to absorbance reduction with rates of 0.001/min was considered one unit of lysozyme activity for each mg of sample.

Alternative complement activity was assessed according to the sheep red blood cell hemolysis (SRBC), following the procedure described by Ortuño et al. (29). The sample volume, which consisted of 50% hemolysis, was set and was utilized to evaluate the complement activity of the samples (value of ACH50 are in units.ml^−1^). A lysis curve was formed on checkered paper to evaluate the quantity of intermediate activity on the complement (Log-Log Graph). Complement activity is calculated using the following equation and based on the amount of serum that caused 50% hemolysis.


$$\begin{array}{l} \text{ACH50 (U/ml)}=\text{k}\times \text{0.5}\times \text{(dilution factor)}. \end{array}$$

K is the amount of serum in ml that yielded 50% hemolysis, 0.5 is constant, and the dilution factor in this experiment was considered 0.01 since the serum was diluted 100 times.

Serum total Ig was measured according to the method presented by Siwicki et al. (30). Protease activity was assessed by the azocasein hydrolysis method as described by Ross et al. (31). Total protein content in samples was assayed by commercial kits (Pars Azmun, Iran) in line with the given instructions. The activity of acid phosphatase was measured using a commercially available kit (Darman Faraz Kaveh, Iran) following manufacturer's instructions.

### Antioxidant Parameters

The serum concentration of superoxide dismutase (SOD), catalase (CAT), glutathione reductase (GR), and malondialdehyde (MDA) were measured by ZelBio kits (Zelbio, Germany) in accordance with the kits' instructions.

### Gene Expression Study

RNA extraction was carried out on intestine and liver samples by RNX-Plus (Sinaclon, Tehran, Iran) according to the manufacturer's instructions. Then cDNA was constructed using 1.00 μg of RNA (Sinaclon, Tehran, Iran) which was preserved at −20°C until further tests.

A 2X SYBR green master mix (Sinaclon, Tehran, Iran) was used to amplify the constructed cDNA in real time PCR (StepOne, Applied Biosystem). In brief, 1 μl cDNA and 0.2 μl primers were added to 5 μl of 2X SYBR green PCR Master Mix and the volume was raised to 10 μl using DEPC-treated water. The real-time PCR conditions are provided in supplementary materials. The primers were designed using Primer3 software based on existing cDNA sequences in the GenBank. The threshold cycle (CT) was determined and normalized to GAPDH as reference gene. The relative expression of selected genes was determined according to Livak and Schmittgen (32) and iQ5 optical system software version 2.0 (Bio- Rad, USA) was used to analyze data.

### Statistical Analysis

The study outline was planned and carried out in an absolute randomized design. After which, statistical analysis of data was conducted. Later inspection of the normality of their distribution by the Kolmogorov-Smirnov test, using one-way analysis of variance (one-way ANOVA) as well as Tukey's HSD *post-hoc* test in SPSS software version 20 was conducted and statistical significance was considered *p* < 0.05.

## Results

### Growth Performance

Rainbow trout growth parameters fed with experimental diets for 45 days are presented in Table 2. Results showed that the individuals from T2 and T3 had a significantly better performance when compared to the control fish (*p* < 0.05). According to the results, the lowest feed conversion ratio and the highest SGR were found in T3. No mortality was observed during the experiment in all treatments. No significant difference was found in WG and SGR values between the control and T1 groups (*p* > 0.05).

**Table 2:** Growth performance of rainbow trout fed diets incorporated with different levels of zero (Control), 0.5% (T1), 1% (T2) and 2% (T3) of a mixture of herbs (*Mentha longifolia, Thymus carmanicus, Trachyspermum copticum*) for 45 days.

**Parameters**	**Control**	**T1**	**T2**	**T3**
Initial weight (g)	22.85 ± 0.99	24.22 ± 1.67	22.59 ± 1.28	22.79 ± 1.34
Final weight (g)	40.82 ± 1/52	42.94 ± 1/74	42.12 ± 2/08	42.57 ± 1.27
Weight gain (g)	17.98 ± 0/92^b^	18.72 ± 1/02^ab^	19.53 ± 0/28^a^	19.78 ± 0.28^a^
SGR (% d^−1^)	1.29 ± 0.02^b^	1.27 ± 0.01^b^	1.38 ± 0.00^a^	1.39 ± 0.01^a^
FCR	1.40 ± 0.07^a^	1.21 ± 0.06^b^	1.17 ± 0.01^b^	1.14 ± 0.02^b^
Survival rate (%)	100.00 ± 0.00^a^	100.00 ± 0.00^a^	100.00 ± 0.00^a^	100.00 ± 0.00^a^

*SGR, specific growth rate; FCR, feed conversion ratio. Data represent mean ± SE. Different letters in the same row indicate significant differences (p <0.05)*.

### Digestive Enzyme Activity

At the end of the dietary experiment, the activity of digestive enzymes, including protease, amylase, and lipase, were measured as presented in Table 3. The highest activity of amylase was found in T1 followed by T2, both significantly different from the control fish (*p* < 0.05). The measured values from T3 were statistically different from the control (*p* < 0.05). However, a reduction in enzyme activity was observed in comparison to the other treatments with a lower inclusion level. Similar results were observed for lipase, with a gradual reduction in enzyme activity as the inclusion level increased. For protease activity, both T1 and T2 were found significantly higher when compared to the control and T3 group. This finding, however, was similar to the results from amylase activity. No statistical difference was found between T1 and T2.

**Table 3:** Digestive enzymes of serum of rainbow trout fed diets incorporated with different levels of zero (Control), 0.5% (T1), 1%(T2) and 2% (T3) of a mixture of herbs (*Mentha longifolia, Thymus carmanicus, Trachyspermum copticum*) for 45 days.

**Parameters**	**Control**	**T1**	**T2**	**T3**
Amylase (U/mg prot)	23.38 ± 0.54^c^	40.56 ± 0.29^a^	40.63 ± 0.49^a^	32.54 ± 0.10^b^
Lipase (U/ mg prot)	1.99 ± 0.05^d^	3.68 ± 0.08^a^	3.10 ± 0.09^b^	2.30 ± 0.04^c^
Protease (U/ mg prot)	6.23 ± 0.07^c^	10.19 ± 0.04^a^	10.36 ± 0.15^a^	8.18 ± 0.04^b^

*Data represent mean ± SE. Means with different letters (a–d) in the same row indicate significant differences (P < 0.05)*.

### Serum Biochemistry

Biochemical parameters measured in fish serum after 45 days of the dietary experiment are presented in Table 4. The highest serum total protein level was present in T2 followed by T1, and all treatments were significantly different from the control. Similar results were also found for albumin. The lowest values were observed in T1 in terms of cholesterol and triglyceride, with no significant difference from the control. Triglyceride in T2 was also statistically different from that of the control. Based on the results, stress biomarkers (glucose and cortisol) were strongly reduced in T1 and T2 compared to the control and T3 groups with no significant difference.

**Table 4:** Biochemical parameters of rainbow trout fed diets incorporated with different levels of zero (Control), 0.5% (T1), 1% (T2) and 2% (T3) of a mixture of herbs (*Mentha longifolia, Thymus carmanicus, Trachyspermum copticum*) for 45 days.

**Parameters**	**Control**	**T1**	**T2**	**T3**
Total protein (g/L)	1.43 ± 0/00^c^	1.77 ± 0.02^ab^	1.85 ± 0/03^a^	1.63 ± 0/08^b^
Albumin (g/L)	0.62 ± 0.01^c^	0.85 ± 0.02^a^	0.86 ± 0.01^a^	0.73 ± 0/03^b^
Triglyceride (mg/dL)	108.00 ± 5.13^a^	65.00 ± 1.15^c^	94.00 ± 2.08^b^	115.00 ± 4.04^a^
Cholesterol (mg/dL)	85.33 ± 4.33^a^	58.66 ± 4.70^b^	79.33 ± 0.88^a^	86.00 ± 1.52^a^
Glucose (mg/dL)	65.66 ± 1.20^a^	44.33 ± 1.85^c^	54.00 ± 2.08^b^	65.33 ± 1.20^a^
Cortisol (nmol/L)	17.60 ± 0/43^a^	16.10 ± 0.35^b^	15.00 ± 0.25^c^	16.63 ± 0.08^ab^

*Data represent mean ± SE. Different letters (a–c) in the same row indicate significant differences (p < 0.05)*.

### Immune Responses

Serum immunological parameters, including lysozyme, alternative complement hemolytic activity (ACH50), total immunoglobulin (total Ig), and acid phosphatase (ACP) activity, were measured (Table 5). Lysozyme activity had increased in all experimental diets compared to the control; however, only T2 was statistically different (*p* < 0.05). Results showed that ACH50 was the highest in T2, quite different from all other treatments. However, with no significant difference, ACH50 in T1 and T3 was higher than the control and lower than T2. Total Ig was found to be augmented in T1, and T2 followed by T3, all of which were meaningfully different from the control group (*p* < 0.05). The activity of ACP was remarkably decreased in T1 followed by T3 and T2, while the highest ACP activity was found in the control group.

**Table 5:** Serum immunological parameters of rainbow trout fed diets incorporated with different levels of zero (Control), 0.5% (T1), 1% (T2) and 2% (T3) of a mixture of herbs (*Mentha longifolia, Thymus carmanicus, Trachyspermum copticum*) for 45 days.

**Parameters**	**Control**	**T1**	**T2**	**T3**
Lysozyme (U/ml)	32.66 ± 0.88^b^	34.00 ± 0.57^b^	40.00 ± 1.52^a^	35.66 ± 0.33^b^
ACH_50_ (U/ml)	135.33 ± 0.33^c^	139.00 ± 0.57^b^	145.33 ± 1.45^a^	138.33 ± 0.33^b^
Total Ig (mg/ml)	8.71 ± 0.03^c^	10.90 ± 0.14^a^	11.29 ± 0.18^a^	10.18 ± 0.0.16^b^
ACP (U/ml)	1.53 ± 0.03^a^	0.87 ± 0.00^d^	0.95 ± 0.00^c^	1.04 ± 0.02^b^

*Ig, immunoglobulin; ACP, acid phosphatase. Data represent mean ± SE. Different letters in the same row indicate significant differences (p < 0.05)*.

### Antioxidant Parameters

The antioxidant parameters, including CAT, SOD, MDA, and GR were determined in serum samples, and results are shown in Table 6. According to the results, the highest level of CAT was in the control group, while SOD was highest in T2. The values for MDA were found to be higher in experimental treatments in comparison to the control (*p* < 0.05), with T2 showing the highest MDA content.

**Table 6:** Serum antioxidant indices and MDA content of rainbow trout fed diets incorporated with different levels of zero (Control), 0.5% (T1), 1% (T2) and 2% (T3) of a mixture of herbs (*Mentha longifolia, Thymus carmanicus, Trachyspermum copticum*) for 45 days.

**Parameters**	**Control**	**T1**	**T2**	**T3**
CAT (U/ml)	70.93 ± 0.26^a^	64.16 ± 0.96^c^	67.46 ± 0.52^b^	69.06 ± 0.86^ab^
SOD (U/ml)	90.30 ± 0.50^d^	92.70 ± 0.49^c^	97.43 ± 0.52^a^	95.43 ± 0.66^b^
MDA (mmol/ml)	63.83 ± 0.92^c^	76.10 ± 0.87^a^	77.53 ± 1.21^a^	72.16 ± 1.29^b^
GR (U/ml)	1063.33 ± 26.66^c^	1277.66 ± 7.31^a^	1250.00 ± 7.63^a^	1156.66 ± 10.72^b^

*Data represent mean ± SE. Different letters in the same row indicate significant differences (p < 0.05)*.

### Skin Mucus Immunological Parameters

**Figure 1:**
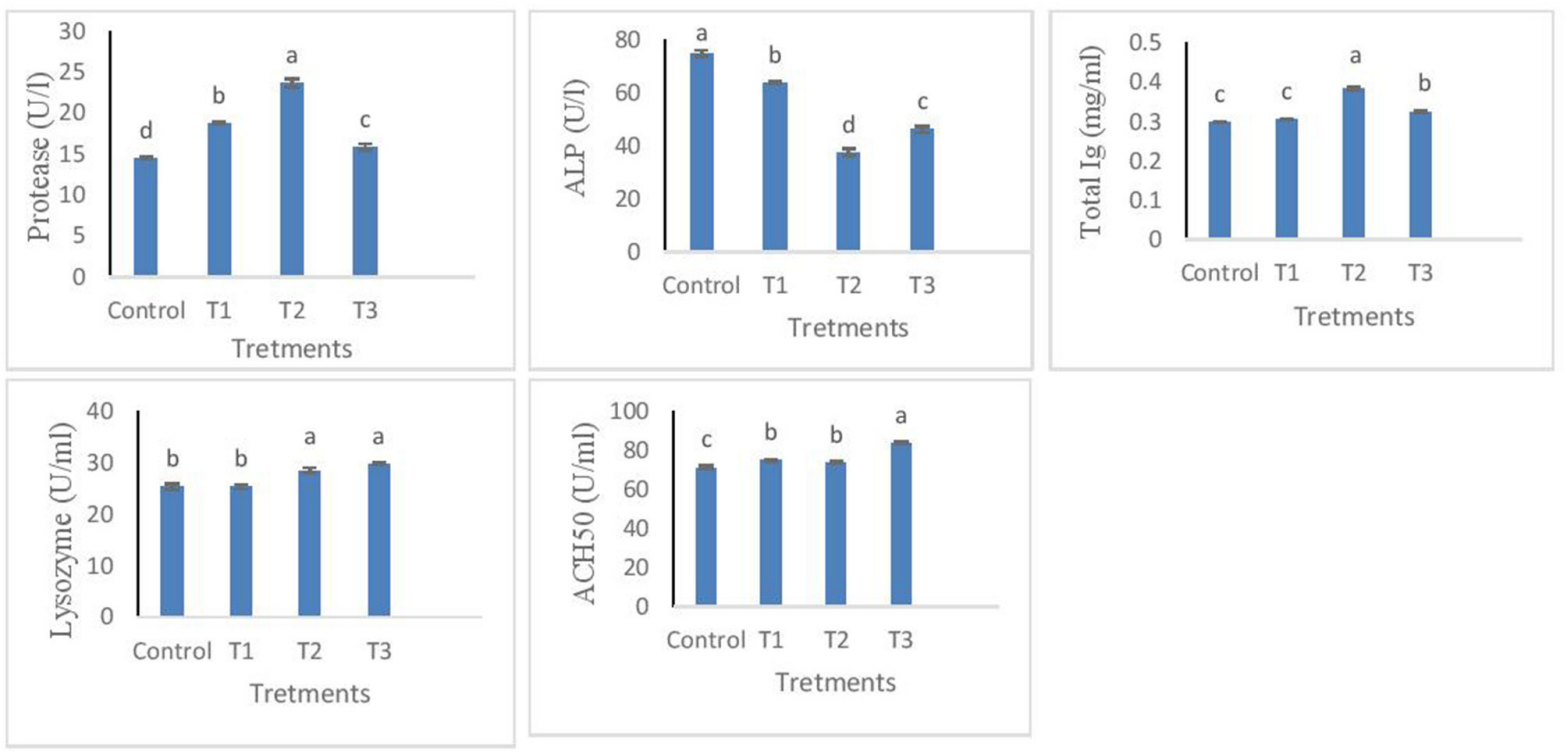
Protease, ALP, total Ig, lysozyme, ACH_50_ levels in the skin mucus of rainbow trout fed diets incorporated with different levels of zero (Control), 0.5% (T1), 1% (T2) and 2% (T3) of a mixture of herbs (*Mentha longifolia, Thymus carmanicus, Trachyspermum copticum*) for 45 days. Data represent mean ± SE. Bars with different letters indicate significant differences (*P* < 0.05).

The results from measured immune responses of protease, ALP, total immunoglobulin, lysozyme, and ACH50 are depicted in Figure 1. Protease was effectively influenced in T2. However, T1 and T3 were also found significantly higher than control (*p* < 0.05). For ALP, T2 showed the lowest value, followed by T3. Total Ig was increased in T1 but not different from control, while T2 and T3 showed a significantly higher value than T1 and control. Similar results were found for lysozyme, except that T3 led to the highest values. In terms of ACH50, individuals from T3 treatment had higher values compared to other treatments. There was no significant difference between T1 and T2, while both were significantly higher than control (*p* < 0.05).

### Gene Expression

**Figure 2:**
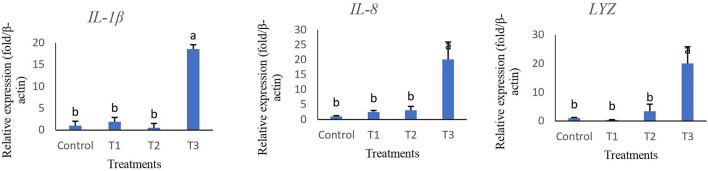
Relative expression of key immune genes of interleukin 1-β (*IL-1*β), interleukin 8 (*IL-8*), and lysozyme (*Lys*) in intestine tissue of rainbow trout fed diets incorporated with different levels of zero (Control), 0.5% (T1), 1% (T2) and 2% (T3) of a mixture of herbs (*Mentha longifolia, Thymus carmanicus, Trachyspermum copticum*) for 45 days. Data represent mean ± SE. Bars with different letters indicate significant differences (*P* < 0.05).

**Figure 3:**
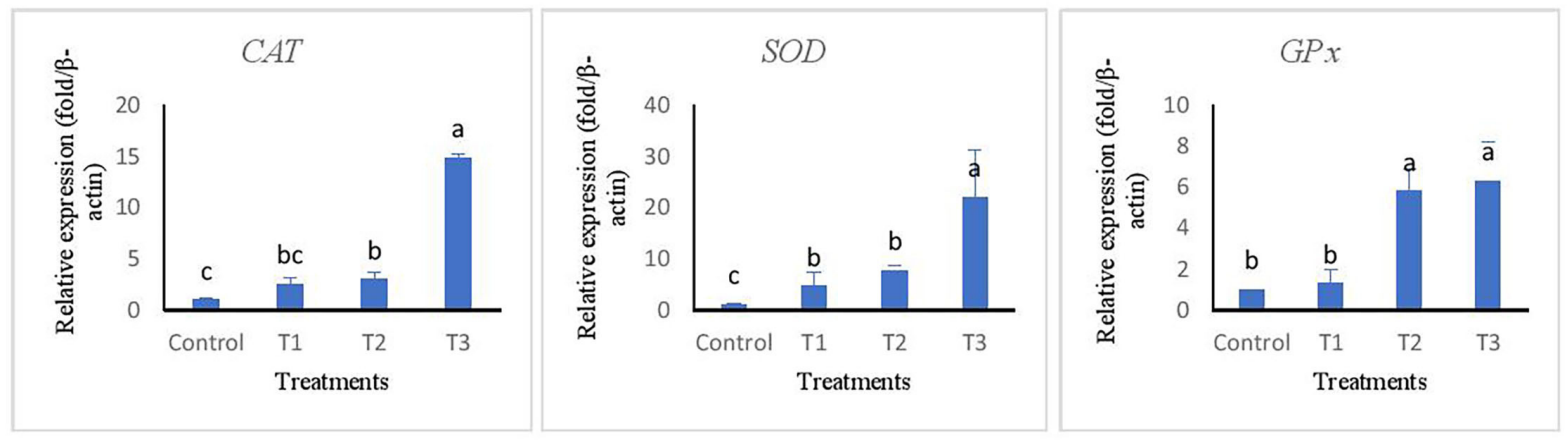
Relative expression of antioxidant related genes of superoxide dismutase (SOD), catalase (CAT), and glutathione reductase (GPx) in liver tissue of rainbow trout fed diets incorporated with different levels of zero (Control), 0.5% (T1), 1% (T2) and 2% (T3) of a mixture of herbs (*Mentha longifolia, Thymus carmanicus, Trachyspermum copticum*) for 45 days. Data represent mean ± SE. Bars with different letters indicate significant differences.

The relative expression of immune and antioxidant genes are represented in Figures 2, 3. Immune-related genes including *IL-1*β, *IL-8*, and *Lys* were significantly up-regulated in T3, while no statistical difference was found when comparing T1 and T2 with the control group (*p* > 0.05). The results also showed that antioxidant genes coding *GPx, SOD*, and *CAT* were highly expressed in T3 and T2 compared to T1 and control groups.

## Discussion

According to the present results, a synergistic effect of *T*. *carmanicus, M. longifolia*, and *T. copticum* (TMT) was identified on rainbow trout growth (weight gain and FCR) and immune response (lysozyme and ACH50 activity). These parameters were found to be highly improved in T2 group. In addition, immune and antioxidant-related genes were modulated by the inclusion of a combination of plants. A notable upregulation was observed for pro-inflammatory genes (*IL-1*β and *IL-8*), as well as *TNF-*α.

The beneficial effects of inclusion of medicinal plants in aquafeeds have been previously reported (2, 3). Although, the exact mechanism of action of medicinal plants on fish growth or immune system is not yet fully understood. It is suggested that these positive effects on animals are due to different bioactive compounds found in medicinal plants including flavonoids, phenolics, alkaloids, saponins, terpenoids, tannins, glycosides, steroids or essential oils (3, 33).

In this study, three plants containing several substances such as carvacrol and thymol have been previously found effective on fish (34, 35). Previous reports testing different levels of carvacrol and thymol suggested their positive effects on growth performance and immunity of rainbow trout (36, 37). Present findings showed better results for growth parameters of WG and FCR in higher inclusion levels of plants (T2 and T3). It suggests a synergistic effect at higher inclusion levels that can be attributed to the major components of the plants. It has been reported that the addition of plant extracts into the fish diet can stimulate appetite (38–40) and increase food intake, which in turn improves growth performance (41). Other researchers have illustrated that combining five different plant extracts improved weight gain in grouper, *Ephinephelus tauvina*, by 41% higher than the control group (42). This finding is similar to the results reported by Ji et al. (43) when fed olive flounder (*Paralichthys olivaceus*) with four different plant extracts.

In the present study, the activity of digestive enzymes was increased in T1 and T2, with a slight decrease in T3. Similar effects have been found for different plant extracts tested on various fish species (44, 45). In a recent study by Ghafarifarsani et al. (28), a combination of medicinal plants was found effective on digestive enzymes regardless of the inclusion level. However, in the present study, the activity of digestive enzymes was decreased in the highest inclusion levels.

The biochemical parameters are reliable indicators of fish general health status. The present results showed that the lower level of incorporation of a mixture of plants exerted the best effects. Stress biomarkers were significantly decreased in T2, which suggests that 1% dietary inclusion was best. This can be further investigated by the combined use of the plants tested in this study as a sedative and/or tranquilizer in aquaculture (46).

Skin mucus functions as the outer layer of the fish defense system which includes several immunological mechanisms such as lysozyme and complement system proteins. Serum immunological parameters are also vital in fish systemic defense capacity. In the present study, all experimental diets containing TMT enhanced both serum and mucosal immune responses in which 0.5 and 1% inclusion levels elicited better immunological responses both in serum and skin mucus. Similar results are available for the positive effects of plant extracts on the immunological responses of fish (8, 9). In a merely similar study, Abdellatief et al. (47) have reported positive effects of combined use of sage (*Salvia officinalis*) and *Spirulina platensis* (*Arthrospira platensis*) on immune responses of Nile tilapia (*Oreochromis niloticus*). The present findings suggested that 1% inclusion level was the most significant on different parameters; however, others have suggested better results associated with higher levels. This might be attributed to the excess of bioactive substances disturbing fish immune and antioxidant systems.

Phytochemicals from medicinal plants have been found to modulate the expression of several genes in fish, which has been reviewed recently by Ahmadifar et al. (48). *IL-1*β is a pro-inflammatory gene released from white blood cells (WBCs), attracting other WBCs and initiating an inflammatory response. In the present study, the transcription of *IL-1*β was significantly increased in the T3 group, likely suggesting that higher levels of incorporation of TMT induced tissue toxicity in fish. However, fish from the T3 group showed a significantly better growth performance when compared to control and other treatments.

IL-8 is another cytokine secreted by a variety of tissues and blood cells involved in attracting neutrophils to the inflammation site and acting as an anti-inflammatory factor. Based on present results, IL-8 was up-regulated in fish receiving TMT. Lysozyme is a critical player in the innate immunity of fish that is coded by a protein-coding gene named *LYZ*. Similar results were found for the *LYZ* gene consistent with increased lysozyme activity in fish receiving TMT.

Zargar et al. (49) have reported a reduction in expression of *IL* and *LYZ* at 2 mL/kg concentration of *Thymus vulgaris* essential oils in the rainbow trout diet. In contrast with present results, a reduction in expression of *IL-1*β and other pro-inflammatory genes was observed when menthol oil was added to the diet of Nile tilapia (50).

Antioxidative-associated genes in liver tissue were also significantly modulated by TMT where the expression of *SOD* and *CAT* was drastically increased in T3, while fish from T1 and T2 showed relatively lower levels. This finding might suggest that fish were under oxidative pressure caused by TMT, in which case MDA levels were also increased in T3. Similar to the present results, 3% fenugreek seed powder resulted in an up-regulation of *IL-1*β and *TNF-*α, reduced levels of AST, and ALT in Nile tilapia (51).

In conclusion, present results suggest that a combination of medicinal plants can be used to enhance the growth and immunity of rainbow trout. Based on gene expression results, it is suggested that a higher inclusion level of a mixture of the plants used in this experiment can trigger inflammatory responses, which is suggested for further research coupled with histological studies. The obtained results in this experiment could be attributed to several present bioactive substances urging further research to identify the main effective substances and their mechanisms of action to modulate immune responses and regulate transcription of critical immune and antioxidant genes.

## Data Availability Statement

The original contributions presented in the study are included in the article/supplementary material, further inquiries can be directed to the corresponding author/s.

## Ethics Statement

The animal study was reviewed and approved by Islamic Azad University of Shahrekord.

## Author Contributions

MR: investigation, data curation, resources, conceptualization, methodology, supervision, and writing and editing. MA, KS, and MM: resources, literature searching, and methodology. GR: writing the original draft and editing. All authors contributed to the article and approved the submitted version.

## Conflict of Interest

The authors declare that the research was conducted in the absence of any commercial or financial relationships that could be construed as a potential conflict of interest.

## Publisher's Note

All claims expressed in this article are solely those of the authors and do not necessarily represent those of their affiliated organizations, or those of the publisher, the editors and the reviewers. Any product that may be evaluated in this article, or claim that may be made by its manufacturer, is not guaranteed or endorsed by the publisher.
